# The Understanding of Pediatric Akinetic Mutism

**DOI:** 10.7759/cureus.12593

**Published:** 2021-01-09

**Authors:** Luiz Severo Bem, Júlia Gemir, Renata R. M Cysneiros, Hildo C Azevedo

**Affiliations:** 1 Neurological Surgery, Hospital da Restauração, Recife, BRA; 2 Neuroscience Post-Graduate Program, Federal University of Pernambuco, Recife, BRA; 3 Neurological Surgery, University of Pernambuco, Recife, BRA; 4 Neurological Surgery, Restauração Hospital, Recife, BRA

**Keywords:** akinetic mutism, pediatric akinetic mutism, pathophysiology, dentate nucleus

## Abstract

Pediatric akinetic mutism syndrome is a clinical disease resulting from cerebellar injury and characterized by the absence of speech or reduced speech, emotional lability, there may also be hypotonia, oropharyngeal dysfunction/dysphagia, bladder and intestinal incontinence, or other behavioral disorders and neurological signals. It is described as the most recurrent complication in children, after posterior fossa tumor surgery, mainly related to cerebellar midline injuries. An increasing number of research and prospective reviews have provided valuable information on cerebellar mutism syndrome in recent years. The purpose of this review was to elucidate the pathophysiological basis and the predictive factors for this syndrome. Most cases of mutism are due to injury cerebellar tracts and cerebellar-cerebral circuits, involving particularly distinct points of the dentate-thalamus-cortical and dentato-rubro-thalamus-cortical. Advanced neuroimaging techniques, such as tractography and perfusion studies, have contributed to demonstrating changes in these pathways in patients with pediatric cerebellar mutism.

## Introduction and background

According to the new studies, the current characteristics of mutism can be defined as delayed onset mutism/reduced speech and emotional lability. Additional features may include hypotonia, oropharyngeal dysfunction/dysphagia, bladder and bowel incontinence, or other behavioral and neurological disturbances [1,2].

Posterior fossa surgical access is one of the most prevalent in children. This access is related to akinetic mutism (AM). Mutism cases are related in the age range from two to ten years. Moreover, it is known as the most common histological type of tumor of the posterior fossa is medulloblastoma [3,4].

Because of the uncertainties regarding the pathophysiological basis of mutism, the main hypotheses have been used over the years. Currently, the involvement of the dentate-thalamus-cortical (DTC) circuit is more accepted, related to disturbance of cerebellum-cerebral. About the mechanism of this disturbance, axons of dentate nucleus leave the cerebellum through the superior cerebellar peduncle, then decussate in the midbrain, reach the thalamus, and, finally project to the cortex (e.g., prefrontal, temporal, and parietal). Damage leading to AM (akinetic mutism) can occur anywhere along the tract. However, given a close relationship with posterior fossa surgery, it is more likely to involve initial parts (e.g., dentate nucleus or superior cerebellar peduncle). The pathophysiological basis of akinetic mutism has not been fully elucidated and there are several controversies [4-6].

Briefly, the goal of this article is to clarify the pathophysiological bases of pediatric akinetic mutism in order to detail its symptoms and its predictive factors.

## Review

Cerebellar mutism (CM) usually develops more in children than adults, after posterior fossa surgery. This condition does not usually appear immediately after surgery but after hours or days. The fact that mutism is a transient condition, it can resolve itself in a period between four and six weeks to a few months. In addition, mutism generates cognitive and behavioral disorders in the patient, and some consequences may persist in view of the persistence of CM [7]. Table 1 provides a summary of articles on the topic in recent years.

**Table 1:** Summary of studies related to pediatric akinetic mutism. PFS: posterior fossa syndrome; AM: akinetic mutism; PPCMS: postoperative pediatric cerebellar mutism syndrome; CSF: cerebrospinal fluid

Author/Year	Objectives	Methods	Conclusions
da Silveira et al., 2017 [8]	Describe and analyze a case report to assess the signs and symptoms that the patient developed with PFS. Case report of one patient.	Case Report.	Case report of an eight-year-old patient with medulloblastoma who developed posterior fossa syndrome after surgical intervention. Therefore, as PFS can cause drastic and eventually disabling changes, and its pathophysiology suggestive of injury to the toothed nucleus and/or its efferents, it is imperative to recognize this condition and exclude other operative complications. That said, the treatment is favorable.
Arnts et al., 2020 [9]	Describe the pathophysiology of the disease and its treatment.	Scientific Article.	AM is a rare but serious neurological disorder. Patients with AM do not have the motivating vector for the expression of the spontaneous mechanism, behavior, and speech. This results in almost complete dependence on others for the simplest activities of everyday life, constantly leading to nursing at home.
Nicita et al., 2016 [4]	Describe a case report of a pediatric patient who developed cerebellar mutism after neurosurgery. In the report, he points out medications for the treatment of this condition. For this, he also seeks to describe the pathophysiology of the disease. Case report of one patient.	Case Report.	The pathophysiological basis of PPCMS has not yet been fully elucidated and there are several controversies and the current hypothesis is that PPCMS is related to cerebral circuit disturbance involving particularly the thalamus-cortical toothed pathway. Concluding the objective of the study, this single patient does not allow to conclude the company, and this study should be replicated in a large sample of PPCMS in the postoperative period in pediatrics and adult to understand whether benzodiazepines can play an effective role in the treatment of this condition.
Tovar-Spinoza and Choi, 2016 [10]	Describe 11 pediatric patients with tumors. One of them presented akinetic mutism and the patient's symptoms. Description of 11 patients, including one with mutism.	Clinical Article.	Patient 7, who had NF1 and thalamic pilocytic astrocytoma of the middle brain, experienced acute edema after severe ablation that caused dense hemiparesis, akinetic mutism, and eye movement disorder. Our hypothesis is that overheating of the 2D target area and the longer ablation time contributed to the conductive heating of the peritumoral tissue. The patient presented concomitant hyponatremia and fluid overload during the operation. Since then, we have carefully monitored these parameters intraoperative period. This patient was treated with greater steroid dosages (1 mg/kg/day) and NaCl at 3%. Steroids decreased two weeks after surgery. The patient showed progressive improvement after rehabilitation and, 18 months after ablation, presented only mild residual weakness in the hands and dysarthria.
Yang et al., 2015 [11]	Describe the case of a pediatric patient with humoral immunity deficiency who presented lethargic encephalitis with isolated lesions of black matter on magnetic resonance imaging followed by the second encephalitis associated with akinetic mutism. Case Report of one patient.	Case Report.	He presented drowsiness, Parkinson's syndrome (facial mask, akinetic mutism, tremor, stiffness), ophthalmoplegia, and nystagmus, meeting the diagnostic criteria for lethargic encephalitis. Laboratory tests showed pleocytosis and a positive oligoclonal band in CSF, consistent with other reports. The lesion of the black substance can inhibit the activity of the cortex, reducing dopaminergic entry into the striated ganglion-thalamus circuit of the cortex, which explains Parkinson’s syndrome in this patient.
Suga et al., 2015 [12]	Describe a case report of a pediatric patient with mumps who contracted mumps encephalitis. It also shows mutism and this condition is described in the report. Case report of one patient.	Case Report.	Symptoms of neurological disturbance varied widely in this case. We considered that akinesia and mutism originated from the lesion of the basal ganglia, while hypothalamic injury caused disturbances in the accommodation of body temperature, blood pressure, and heart rate.
Kamas̄ak et al., 2015 [13]	Description and study of bilateral paramedical thalamic infarctions and their involvement in pediatric patients. Two patients were analyzed.	Clinical Observations.	Bilateral paramedian thalamus infarctions can result in many diseases and damage. Common clinical manifestations include disorientation, confusion, hypersolence, deep coma, and "coma wake", or akinetic mutism (lack of response), in addition to severe memory impairment.
Pinheiro et al., 2012 [14]	The article reports on a case of transient cerebellar mutism in a four-year-old child resection of an ependymoma from the posterior fossa.	Case Report.	This case adds to the few hitherto reported cases in the literature of this unusual complication of the subsequent fossa surgery in children.

After surgical procedures, edema and regions of ischemia in organs are normal due to manipulation and trauma caused by the surgery. Thus, it is justified the latency of mutism to manifest, usually taking 24-48 hours to appear the first symptoms. Furthermore, those edema and ischemia would be located in certain regions of the brain that would trigger the symptoms of akinetic mutism [15].

Over the years, studies concerning the pathophysiology of akinetic mutism have been modified. The medical literature has presented many studies addressing the origin of cerebellar mutism. Thus, researches involving the affected dentate nucleus and the complex involvement of the vermis region have been widely discussed and validated in medical sciences for years. However, nowadays, the dentato-thalamo-cortical tract can better elucidate the pathophysiology of the syndrome, being the most accepted study today.

Initially, it was believed that the development of this condition was due to the involvement of the dentate nucleus. After some studies, it was understood that isolated lesions, in this context, the cerebellar ones, would not be able to provoke all the symptoms of akinetic mutism. After discarding the previous hypothesis, it was understandable to look for lesions on both sides of the cerebellum; however, after studies, mutism would be triggered, even if there was no bilateral cerebellar lesion [16].

Later studies associated mutism with lesions in the vermis region, without necessarily having bilateral cerebellar lesion. A fact evidenced in a case study, in which CM that was associated with atrophy of the vermis on MRI and decreased blood flow in the same region on the SPECT (single photon emission computed tomography) scan, and suggested that CM resulted from injury to the vermis with or without a bilateral cerebellar hemisphere lesion [17,18].

Other studies used to believe that the vermis would be the main reason for the symptoms associated with mutism. Nevertheless, if this region were really the only one responsible for the development of CM, this condition would be much more incident and more persistent, which is not true, since the acute manifestations of the disease do not last more than months. This theory was discarded, transferring the reasoning to the possible hypothesis that mutism is not triggered due to the involvement of a single area of the brain, but of a set of brain areas [19,20].

So today, one of the most accepted and persistent theories about the pathophysiology of CM involves the dentato-thalamus-cortical tract. The anatomophysiology of this tract can be described through the course to and from the dentate nucleus of the cerebellum in any region, travels through the superior cerebellar peduncle, crosses the midline to the opposite part within the decussation of the superior cerebellar peduncle, continues through the brainstem to the contralateral ventrolateral nucleus of the thalamus and later to the contralateral cerebral motor cortex (premotor and supplementary motor cortices). The manifestations resulting from mutism are caused by injuries in the course of that tract. When we relate these structures to the posterior fossa surgery, the one that most causes CM in patients, it is possible to notice that proximal parts of the treatment are most affected. Thereafter, regions such as the dentate nucleus, cerebellar peduncles, and/or brainstem connections are closely correlated with the pathophysiology of akinetic mutism [21].

The injuries along the DTC tract cannot be just anatomical injuries along the tract. So, it was necessary to look for another mechanism to justify the involvement of CM symptoms. Thus, cerebellum-cerebral diaschisis can elucidate the pathophysiology of mutism, since this diaschisis corresponds to the functional inhibition of a certain region of the brain, in which that region is connected, through transsynaptic neural pathways, to the area of ​​the primary lesion. It can justify the appearance of symptoms characteristic of brain regions that were not even surgically injured initially [22].

Some hypotheses can confirm this theory, since these cerebellar areas were subjected to imaging techniques that prove the relative loss of physiological function in the affected region, without necessarily having visible damage. It is possible to infer that the lesions may appear before or after the surgery, in which the edema and the vasospasm during and after the surgical procedure, can cause damage to the adjacent venous circulation. Therefore, AM can persist in its clinical presentation until the edema and vasospasm are resolved [23,24].

On the basis of these theories, visible radiological ends up in some studies, a study presented results that were found in patients who developed the post-operative cerebellar mutism syndrome. Post-operative brain MRI usually shows signal abnormalities within the superior cerebellar peduncles and dentate nuclei: T2 - hyperintense signal within the superior cerebellar peduncles and dentate nuclei. Furthermore, MR tractography - can show disruption of the dentate-thalamus-cortical tract [25,26].

Trasnvermian approach is a common route of access to fourth ventricle tumors but with the risk of important morbidity. Several approaches have been recognized to reduce morbidity; some of which include transforaminal, subtonsillar, telovelar approaches, etc. These approaches are devised on the basis that accurate dissection along the natural avascular planes will avoid lesion to the significant structures in this location minimizing morbidity. Therefore, a clear understanding of the posterior fossa anatomy was supposed to facilitate dissection and provide better outcomes. Yet, a significant incidence of postoperative mutism is still seen with this approach in large tumors. One theory could be that because the mutism is a disturbance of cerebellum-cerebral circuitry particularly involving the dentato-thalamus-cortical, and not only from a compromised area of the brain [27,28].

## Conclusions

The pathophysiological basis of postoperative pediatric cerebellar mutism syndrome (PPCMS) is better correlated nowadays with the involvement of the dentato-thalamus-cortical tract. Even with the evolution of microsurgical technique, mutism is still a condition that continues to affect children who perform these surgeries and posterior fossa surgery. Selection of the less risky surgical approaches could be reached by elaborated studies of advanced neuroimaging and solid knowledge of the surrounding anatomy.
